# Lymph Node Dissection for Upper Tract Urothelial Carcinoma: A Critical Appraisal of Lymph Node Mapping Studies

**DOI:** 10.3390/cancers17233843

**Published:** 2025-11-29

**Authors:** Jesse Persily, Simon White, Katie Murray

**Affiliations:** Department of Urology, NYU Langone Health, New York, NY 10016, USA; simon.white@nyulangone.org (S.W.); katie.murray@nyulangone.org (K.M.)

**Keywords:** upper tract urothelial carcinoma, lymph node dissection, retroperitoneum, retroperitoneal lymph node dissection, pelvic lymph node dissection, lymph node template, lymph node mapping, radical nephroureterectomy

## Abstract

Despite the advent and growth of endoscopic and nephron-sparing management approaches, the mainstay treatment for upper tract urothelial carcinoma (UTUC) in 2025 remains radical nephroureterectomy (RNU). Classic teaching, largely derived from the benefit seen in the muscle-invasive bladder cancer (MIBC) population, supports the inclusion of retroperitoneal lymph node dissection (LND), particularly for high-risk and high-grade disease. However, no level 1 evidence exists supporting the inclusion of an LND at the time of extirpative surgery for UTUC. Moreover, studies attempting to map lymph node dissection relative to primary UTUC tumor location are plagued by limitations. Herein, we summarize and review available data regarding proposed LND templates for the management of UTUC.

## Simple Summary

Several clinical practice guidelines recommend the inclusion of a lymph node dissection during the surgical management of tumors of the upper urinary tract (the kidney drainage system), but there is limited evidence supporting the ideal extent of the lymph node dissection that should be performed to optimize patient outcomes. In this review, we summarize the available evidence and highlight key limitations in the literature.

## 1. Introduction

Upper tract urothelial carcinoma (UTUC) refers to urothelial tumors that arise outside along the urinary tract, excluding the bladder. UTUC is far less common than urothelial carcinoma (UC) of the bladder, representing only 5–10% of all urothelial cancers [1]. Though these are related cancers, it has become clear they are unique biologic entities with some overlapping and some unique pathogenesis, clinical behavior, and patterns of spread [1,2,3]. Given the relative rarity of UTUC, much of its management paradigm has been derived from the bladder UC literature, but given the unique nature of UTUC, critical appraisal of the available literature is imperative to ensure the best possible patient management and outcomes based on available data.

Despite advances in endoscopic tools and technology that have in part led to, and in many ways bolstered, endoscopic and nephron-sparing approaches to the management of UTUC, the gold-standard treatment remains radical nephroureterectomy (RNU) with lymph node dissection (LND). This combination is supported by both the most recent European guidelines on the management of UTUC and was similarly supported by the first ever American Urologic Association UTUC guidelines, published in 2023 [1,4]. However, both guidelines highlight the paucity of level one evidence supporting the use of LND at the time of RNU and provide even less data or details regarding the nature of the lymph node dissection that is to be performed. Similarly, the 2025 NCCN guidelines on bladder UC and UTUC state as a surgical principle that a “regional lymph node dissection should be performed for high-grade tumors”, without citing any evidence or defining what the regional lymph node dissection should entail [5]. Additionally, with the recent randomized trial in bladder UC supporting a de-escalation of the extent of the LND performed at the time of radical cystectomy, evaluation of the UTUC LND literature is warranted [6].

In this review, we summarize the available literature regarding the LND performed at the time of extirpative surgery for UTUC, focusing on the limitations of the existing data. Though there is limited data on this specific topic, we performed a narrative review of the PubMed database, focusing on articles that specifically looked at the extent and location of lymph node metastases in UTUC. Articles were included if they reported both the location of the tumor and the location of positive lymph nodes by potential lymph node landing zones. Though some data will be mentioned, the focus of this article is not on whether LND improves outcomes. We will instead highlight the ways in which the limitations of the LND template literature influence our ability to answer questions about the impact of LND on oncologic and survival outcomes. The controversy surrounding whether LND impacts outcomes or simply shifts patient stage has been reviewed previously, and on-going work in large international UTUC studies continues to try to answer this question [7,8].

## 2. What Is the Standard Lymph Node Dissection Performed for Upper Tract Urothelial Carcinoma?

Though LND in bladder UC is not without controversy, there is little question about the propensity for the disease to spread via bilateral pelvic lymph node basins, UTUC lymphatic drainage is less straightforward due to the complexity of ureteral vascular anatomy, and thus nodal metastasis deposition has been more challenging to study [7]. This is due in part to the rarity of the disease and additionally to the wide range of possible tumor locations. This is further complicated by the possibility of multifocal disease, which can be multifocal unilateral or even bilateral. Thus, potential nodal basins in these patients include the renal hilar lymph nodes and retroperitoneal lymph nodes, as well as pelvic lymph nodes. However, given the morbidity of performing even a unilateral full template dissection that encompasses all these areas, efforts have been made to map lymph node metastasis relative to primary tumor characteristics, to provide guidance for surgeons performing lymph node dissection at the time of RNU.

### 2.1. Proposed Lymph Node Dissection Templates

Two classic mapping studies have led to the proposed LND templates for UTUC. The first was a study conducted by Kondo and colleagues, who published their initial mapping study in 2007, followed by longer-term outcomes in 2014 and additional oncologic outcomes in 2017 [9,10,11,12]. The group divided primary tumor location into the right/left renal pelvis and the upper, middle, and lower ureter. They used the IMA and the crossing over the common iliac to subdivide these areas. They did include multifocal tumors and used the tumor with the highest T stage as the primary tumor.

Important for study interpretation is the fact that they used both imaging and final pathology to determine nodal basins. Specifically, a lymph node of 1cm or more was considered metastatic disease. When a lymph node dissection was performed, their standard template was the ipsilateral retroperitoneal (para-caval on the right, para-aortic on the left), as well as the ipsilateral hilar lymph nodes for renal pelvis, upper ureter, and middle ureter tumors and the ipsilateral pelvic lymph nodes for lower ureteral tumors. In some but not all patients, retrocaval and interaortocaval lymph nodes were also removed. Notably, not all patients underwent an LND. To account for these inconsistencies, the authors chose to use several proxies for positive lymph nodes, including clinically positive nodes on imaging and nodal growth after RNU.

This design had several key flaws. It did not allow for the assessment of skip or crossover lesions and may have resulted in the inclusion of “positive” lymph nodes that were in fact inflammatory or reactive in the setting of large primary tumors but were not themselves sites of metastatic disease. The authors themselves highlight this, in that not all patients whom they considered metastatic were found to have positive lymph nodes on final pathology. These authors also used on-going growth of unresected lymph nodes as a proxy for positive lymph nodes.

With these limitations in mind, in their initial cohort, out of 181 patients treated with RNU, 42 (23.2%) met some criteria for having positive lymph nodes in at least one nodal basin and were included in the analysis. Nodal involvement was 20–30% for tumors of the renal pelvis, upper ureter, and middle ureter and roughly 10% for tumors of the lower ureter. The authors then broke these findings down by site of nodal metastasis (Table 1).

**Table 1:** Lymph node metastasis distribution among available upper tract urothelial carcinoma mapping studies.

	Kondo et al. [12] N = 42 ^1^	Matin et al. [13] N = 73 ^2^	Bobjer et al. [14] N = 23 ^1^
Right renal pelvis tumors	Hilar: 44.4%; para-caval 27.8%; retrocaval: 27.8%	Hilar: 22.1%; para-caval: 44.1%; retrocaval: 10.3%; interaortocaval: 20.6%; unspecified: 2%	Para-caval: 50%; interaortocaval 3.3%; para-aortic: 9%; pelvic: 4.5%
Right proximal ureteral tumors	Retrocaval: 50%; interaortcaval: 50%	Hilar: 46.2%; para-caval: 4.2%; retrocaval: 7.7%	
Right mid-ureteral tumors	Retrocaval: 33.3%; interaortocaval: 66.6%	N/A	
Right distal ureteral tumors	Common iliac: 50%; obturator: 50%	Para-caval: 50%; external iliac: 50%	N/A
Left renal pelvis tumors	Hilar: 50%; interaortocaval 5.6%; para-aortic: 44.4%	Hilar: 53%; para-aortic: 31%; interaortocaval: 4%; duprahilar: 1%; common iliac: 1%; aortic bifurcation: 1%; retrocrural: 2%; unspecified: 7%	Suprahilar: 7.4%; interaortocaval: 3.7%; para-aortic: 81.5%; pelvis: 7.4%
Left proximal ureteral tumors	N/A	Hilar: 36.4%; para-aortic: 63.6%	
Left mid-ureteral tumors	Para-aortic: 100%	Hilar: 40%; para-aortic: 40%; internal iliac: 20%	
Left distal ureteral tumors	Common iliac: 50%; internal iliac: 50%	Para-aortic: 33.3%; common iliac: 33.3%; external iliac: 16.7	N/A

^1^ Percentages are positive nodes found in each of the pre-specified nodal basins. An individual patient could have more than one positive nodal basin. ^2^ Percentages are the percent of discrete lymph node metastases in that subset of patients.

The authors’ key finding was that, contrary to the popular belief at the time, the para-aortic and para-caval nodes were not necessary the primary site of metastasis for renal pelvis/upper ureteral tumors. Consistent with this popular belief was the fact that lower ureteral tumors do seem to metastasize to nodal basins at the common iliac node and below, but it is notable that they found nodes at each of the common iliac, obturator, and internal iliac, with no nodes found above the aortic bifurcation when the tumor was in the lower ureter. This supports the common practice of performing a pelvic lymph node dissection at the time of nephron-sparing distal ureterectomy and ureteral reimplant for distal ureteral tumors but highlights that, if the procedure is being performed for staging purposes and/or to help with adjuvant therapy decision making, a full standard pelvic lymph node dissection may be necessary. The authors then conclude that LND should be performed at sites where the incidence is 30% or more. It is unclear from the manuscript where this cut-off originated, and it is not paired specifically with outcome data.

The same group then set out to prospectively validate their proposed templates. The first report was published in 2014, describing 77 patients who underwent RNU and LND. They took the step of comparing outcomes in this group to a group of 89 patients who underwent RNU but did not undergo LND during the study period. The difficulty, as will become clear with all studies of LND in UTUC, is that the overall number of positive lymph nodes discovered was very low—2 patients with renal pelvis tumors and 6 patients with ureteral tumors were found to have pathologically positive lymph nodes in this group [9]. Thus, template validation in a prospective manner remains a key goal in the field.

The second widely cited mapping study was published by Matin and colleagues in 2015 [13]. The group looked at 73 patients across several institutions who were found to have pathologically positive lymph nodes at the time of surgery. Their cohort did include a subset of patients who underwent segmental ureterectomy, as well. They similarly annotated the tumor location, dividing the primary upper tract tumor into the following locations: the renal pelvis (defined as the renal calyces down to the ureteropelvic junction); proximal ureter (defined as the ureteropelvic unction down to the inferior mesenteric artery); mid-ureter (the inferior mesenteric artery down to the iliac vessel); and distal ureter. They took the step of grouping tumors into three categories: renal pelvis +/− proximal ureteral tumors; proximal and/or mid-ureteral tumors only; and distal ureteral tumors only. Patients with renal pelvis and proximal ureteral tumors underwent as a minimum a template dissection of the great ipsilateral vessels. Notable differences between this study and the Kondo study included the fact that their right-sided template always included retrocaval nodes. In the Matin paper, interaortocaval and common iliac node dissection was performed when suspicious nodes were identified in these regions on preoperative imaging. Mid-ureteral tumors underwent para-caval or para-aortic LND, as well as ipsilateral common and external iliac LND, while distal tumors underwent standard ipsilateral pelvic lymph node dissection.

The details of this team’s findings are also included in Table 1. To highlight some key novel findings, the group found that tumors of the mid- and distal ureteral would occasionally manifest the upward migration of metastases to the para-caval and para-aortic regions. Given the interconnected nature of the ureteral vascular and lymphatic anatomy, this finding is not necessarily counterintuitive, but it was the first published occurrence of this finding, and thus, the authors felt that further studies would be needed to elucidate this pattern of spread. In contrast, they also found only a single example of an interaortocaval node in a patient with a right-sided primary tumor. The authors concluded that this is mostly affected only secondarily when patients have right-sided primaries. However, this could also be interpreted as the idea that, even though rare, skip metastases are possible clinically suspicious nodes, even in the absence of lesions in earlier adjacent nodal basins, they likely should not be ignored.

Though in some ways an improvement upon the original mapping study, this study too has limitations. Given its retrospect nature, it is unclear what criteria drove the decision to perform lymph node dissection. Even though there is evidence that imaging inadequately stages UTUC, the Kondo study did attempt to account for lymph node metastases in patients who did not undergo LND, while the Matin study did not. Other small criticisms include possible differences in lymph node dissection templates between the three institutions included in the study, as well as the inclusion of patients with a history of cystectomy, which may have altered both the lymphatic drainage in the region and the available lymphatic tissue for dissection. Finally, though we have reiterated the disease’s rarity and the concomitant low numbers, these studies highlight that most tumors are found in the proximal ureter or renal pelvis, and thus, tumors of the mid- and distal ureter are even more poorly represented. Further complicating the results is that these tumors are often but not always managed with a different operation (distal ureterectomy and ureteral reimplant). Thus, the available data become even more heterogenous.

Despite the very low numbers (42 patients in one study, nearly half of whom did not have confirmed lymph node metastases, and 73 in the other), these studies formed the basis for recommendations from several guidelines panels to include template lymph node dissection [7]. At the time, the 2023 AUA guidelines acknowledged that to date, there was no study with adequate evidence to specifically support this template, but the findings of these studies are consistent with theoretical lymphatic drainage patterns, which, paired with the clinical practicality of the proximity of these areas to the dissection being performed for the RNU or DU, are the templates that should be used in practice.

### 2.2. Updated Lymph Node Mapping Study

A more recent mapping study performed by Bobjer and colleagues was published in 2023 and became the primary source used to justify the need for template-based LND in the most recent iteration of the EAU UTUC guidelines [14]. A few key elements should be highlighted about this study. In contrast to the previously mentioned studies, the more recent nature of this publication meant that both imaging and surgical techniques employed more closely approximate modern-day surgical and imaging principles, including the more common use of minimally invasive surgery. Additionally, and importantly, this was a prospective study, but the authors chose to only include patients with clinical suspicion for nodal disease or clinical suspicion for locally advanced disease, to enrich for nodal positivity, but they did not only include patients who were found to have clinically positive nodes as was the case in the Matin study. It should be noted that in contrast with the prior studies, these authors include only patients with UTUC in the renal pelvis and/or proximal ureter, above the level of the inferior mesenteric artery.

All patients received a full unilateral retroperitoneal lymph node dissection on the side of the primary tumor, as defined for the template dissection for testicular cancer but adjusted to fully include the hilar nodes on each side [15]. Thus, these patients received a more extensive lymph node dissection, particularly those with right-sided tumors, than the previous two studies. Similarly to the Matin studies, additional out-of-template areas could be dissected if there was intraoperative or preoperative concern for nodal involvement in these regions. Both open and robotic approaches were allowed, all patients underwent a preoperative CT or MRI, and screening for metastases was performed via a chest CT or FDG-PET CT. Finally, in contrast to the prior two studies, all patients underwent RNU, with no patients undergoing segmental ureterectomy. This is consistent with the inclusion of only those with proximal tumors.

In total, the investigators screened 114 patients and, after exclusions, 100 patients underwent RNU and LND. However, lymph node metastases were only found in 23/100 patients; 19/23 had pN2 disease, and 7/23 had received neoadjuvant chemotherapy. The average lymph node yield at the time of surgery was 12. Lymph node metastases were found in 11/38 right-sided tumors and 12/62 left-sided tumors. It is notable that the lymph node metastasis rate was still low, even in a cohort that was felt to be enriched for patients at risk for nodal metastases. This highlights the challenges of image-based staging, even in the modern era [16].

The study by Bobjer et al. [14] does provide some updated details about the morbidity of LND in the modern era, in that they report on 100 patients, all of whom received LND. In total, 13 patients had a complication of Clavien grade ≥ 3. Two patients required interventions for complications thought to be directly related to the lymph node dissection—one patient underwent surgical exploration for a persistent lymph leak, and one patient had a percutaneous drain placed for an infected lymphocele. A large-scale, international cohort of robotic-assisted RNU and LND also suggests that the additional of LND remains safe with minimal added risk in the robotic surgery era [8].

This group found similar results to those found overall to the previous studies, in that most positive lymph nodes were found in the right- and left-sided hilar and para-caval or para-aortic templates, respectively. A few notable exceptions were found. One patient with a right-sided tumor was found to have a right pelvic lymph node, one an interaortocaval lymph, and one a para-aortic lymph node. Similarly there were five patients with lymph node metastases outside the pre-specified template for patients with left-sided tumors: one interaortocaval, one suparhilar on both the left and right, and two in the left pelvic region. These findings in and of themselves are not groundbreaking, in that they serve to highlight the complex lymphatic drainage of the ureter. Thus, it is not overly surprising that some proximal ureteral tumors will develop pelvic lymph node metastases, depending on the way the ureteral vascular flow travels in relation to the exact position of the tumor.

However, the authors did highlight another finding that further complicates the LND story. In addition to mapping positive lymph nodes at the time of surgery, they mapped the location of lymph node recurrences and found that regardless of the side of the primary tumor, there was a risk of contralateral and out-of-field lymph node recurrence on follow up. This is highlighted by the outcomes described for these patients. During the follow up period, 10/77 pN0 patients (13%) died of urothelial carcinoma while 14/23 pN1–2 patients (61%) died of urothelial carcinoma, with 18/23 (78%) pN1–2 patients developing urothelial carcinoma recurrence. These findings are consistent with the literature, in that patients with nodal positivity have worse outcomes [17,18,19]. With the information currently at our disposable, it is difficult to say whether the removal of these additional positive lymph nodes outside the template would have improved clinical outcomes, especially for patients who already underwent an extensive, standardized unilateral template dissection. That said, we have summarized the reccomended lymph node dissection templates based on location of primary tumor in Table 2.

**Table 2:** Comprehensive lymph node dissection templates per primary tumor site.

Tumor Location	Suggested Lymph Node Dissection Template
Right renal pelvis tumors ^1^	Hilar and para-caval +/− interaortocaval
Right proximal ureteral tumors ^1^	Hilar, para-caval/retrocaval, interaortocaval
Right mid-ureteral tumors ^1^	Hilar, para-caval/retrocaval, interaortocaval
Right distal ureteral tumors	Pelvic ^2^ +/− para-caval
Left renal pelvis tumors	Hilar, para-aortic, rarely suprahilar/interaortocaval
Left proximal ureteral tumors	Hilar, para-aortic
Left mid-ureteral tumors	Hilar, para-aortic, +/− pelvic
Left distal ureteral tumors	Pelvic +/− para-aortic

^1^. Right-to-left spread resulting in para-aortic metastasis is possible, though rare. ^2^. External iliac, hypogastric, obturator nodes. Exact extent of necessary pelvic lymph node dissection remains unclear.

## 3. Additional Key Limitations of the Available Literature

Though limitations of the individual studies have been highlighted, some additional limitations must be addressed. First and foremost, as is clear from Table 1, the literature itself is still very limited and heterogeneous. The three available mapping studies all present their lymph node metastasis data in different ways, with some emphasizing the number of positive lymph nodes and some instead focusing on the number of patients. The Kondo study, which is the more highly cited, reports on some patients who were not actually found to have lymph node metastases at the time of surgery.

This heterogeneity, even among the studies that were attempting to map lymph nodes, does highlight another key limitation, which is that even now, there is likely significant heterogeneity in the LND performed at the time of RNU in both the literature and practice, and thus, studies that attempt to draw conclusion about patients who undergo LND vs. not should be critically appraised regarding the quality and extent of the LND performed. Though various attempts have been made to do this—by looking at overall lymph node yield or by focusing on the location of eventual nodal recurrences, standard reporting of lymph node dissection outcomes (the number of basins dissected and number of nodes as a starting point) is key in future comparisons.

Even with the addition of the more recent mapping study, very few reports of lymph node metastases include patients with tumors of the lower third of the ureter. The recommendation to perform an ipsilateral pelvic lymph node dissection may seem insignificant, given the ease of performing this step of the operation after the ureter has been sufficiently mobilized, but given the known risk of lymphocele and resultant complications, additional work must be undertaken to better document and understand patterns of lymphatic spread in these tumors. Studies of this nature may also offer insights into which patients would most likely benefit from a nephron-sparing segmental ureterectomy and ureteral reimplant and who might derive more benefit from a more extensive extirpative operation and possibility even a more extensive LND.

Along the same lines, the mapping studies available do not delve into the impact of tumor multifocality on lymph node status at the time of surgery. One recent report by Milojevic et al. focused broadly on the prognostic impact of tumor multifocality but did mention that in patients with multifocal tumors, lymph node status was still a predictor of survival [20]. Though as a clinical principle it would make sense to approach the LND relative to the tumor that is being acted upon, which likely represents the highest-risk lesion, data supporting this approach does not yet exist. Future mapping studies could evaluate the rate and risk of out-of-field metastases in these patients.

Additionally, it is unclear how the increased utilization of endoscopic management of ureteral and renal pelvis tumors will influence these outcomes when patients do eventually require extirpative surgery. Particularly if patients with initially low-grade tumors progress after several attempts at nephron-sparing procedures, it seems that concomitant periureteral fibrosis or possibly even altered ureteral blood flow secondary to endoscopic intervention could influence lymphatic drainage and thus metastatic sites, though this is speculative at this time. Though tumor grade seems to have emerged as a useful risk stratifier, clinically or pathologically staging ureter and renal pelvic tumors remains a challenge, given the difficulty in obtaining interpretable pathologic samples that demonstrate the depth of invasion [21]. An inability to accurately assess T stage may then influence the treatment decision or decision to proceed with LND and subsequently negatively impact patient outcome. This too is an area that needs further investigation.

It is worth considering that if the cohort of patients undergoing RNU and LND is higher-risk/higher-stage because of lower-risk patients being managed endoscopically, outcomes may not be comparable to historic standards. This variation in the so-called “Will Rogers” phenomenon, however, may enrich the surgical UTUC for patients who are most likely to benefit from LND, but on-going investigations into who derives the most potential benefit remain to be performed [22]. As we have highlighted, one of the major benefits of LND seems to be improved lymph node staging compared to conventional or PET imaging, though there is increasing evidence that PET imaging may have a sensitivity as high as 82–95% for clinically detecting node-positive disease [23]. If, in the future, there is novel imaging that significantly improves the accuracy of preoperative staging, the risks of LND may begin to outweigh the benefits. As with muscle-invasive bladder cancer, with the increasing use of enfortumab-vedotin with improved pathologically complete response rates, whether patients benefit from consolidative surgery or can continue with active monitoring in the absence of clinically obvious disease remains unclear [24,25]. Accurate pre- and post-treatment imaging may play a key role in selecting patients for neoadjuvant or adjuvant treatment or consolidative surgery, and what role LND will play in these patients remains unclear as well.

Finally, as discussed previously, the classic mapping studies were performed prior to the era of minimally invasive surgery. Even the more recent Bobjer study only included six robotic procedures [14]. Utilization studies suggest that this has been on the rise for the last 15 years in the United States, and reports from a large international cohort have begun to be published, but the overall impact of the robotic approach on adequate nodal staging and the resultant outcomes remains to be fully determined [19,26,27,28,29]. It is important to consider how the surgical approach influences adverse events, the surgeon learning curve, and, in the case of the current discussion, whether the robotic approach positively or negatively impacts surgeon decision making regarding performing an LND and the extent of the LND performed. There is increasing evidence to suggest that the robotic approach does not negatively impact oncologic outcomes, and preliminary studies have shown that short-term complications appear to favor the robotic approach, but studies focused on minimally invasive LND specifically for UTUC are lacking [30,31]. Several studies have included these patients along with patients undergoing LND for testicular cancer via a minimally invasive approach and have shown favorable short-term outcomes and relatively low complication rates, but prospective studies are needed [32,33].

## 4. Conclusions

UTUC is a unique, heterogenous, and challenging entity whose management continues to evolve. Despite several guidelines and statements recommending the performance of an LND at the time of extirpative surgery, the literature defining attempts to perform LND is limited. UTUC does appear to spread in a predictable, albeit not entirely consistent, pattern, and further work is needed to map the frequency and location of nodal metastases relative to a wide range of primary tumor characteristics. This will in turn allow better comparison, better staging, and, hopefully, ultimately better risk stratification and patient outcomes.

## Abbreviations

The following abbreviations are used in this manuscript:

UTUC	Upper Tract Urothelial Carcinoma
RNU	Radical Nephroureterectomy
LND	Lymph Node Dissection
UC	Urothelial Carcinoma
EAU	European Association of Urology
DU	Distal Ureterectomy
SU	Segmental Ureterectomy
MIBC	Muscle-Invasive Bladder Cancer
